# Preclinical assessment of inhalational subacute safety of nebulized MP-171 (BromAc®)

**DOI:** 10.1186/s12890-025-04090-1

**Published:** 2026-01-06

**Authors:** Ahmed H. Mekkawy, Md Khalilur Rahman, Krishna Pillai, Samina Badar, Mirela Simic, Javed Akhter, Sarah J. Valle, David L. Morris

**Affiliations:** 1 Mucpharm Pty Ltd., Sydney, NSW 2217 Australia; 2 Department of Surgery, St George Hospital, Sydney, NSW 2217 Australia; 3 St George & Sutherland Clinical School, University of New South Wales, Sydney, NSW 2217 Australia; 4 Intensive Care Unit, St George Hospital, Sydney, NSW 2217 Australia

**Keywords:** BromAc^®^, Cytokines, Preclinical, Respiratory, Extended safety

## Abstract

BromAc^®^ (MP-171) is a novel first in class therapeutic agent under investigation for its potential in treating respiratory diseases. Evaluating its safety profile following inhalation is necessary for future clinical application. Here, we evaluated BromAc^®^ safety in an extended subacute short-term inhalation study. Forty BALB/c mice were divided into five groups (*N* = 8 each): sham control, saline control, and three BromAc^®^ dose groups at 0.250/20, 0.500/20, and 0.750/20 mg/mL. Treatments were administered by inhalation three times daily for 28 days. A final single dose was given on Day 29 before euthanasia. Various parameters were assessed during the study to evaluate the effect of BromAc such as wellbeing and bodyweight, along with tissue pathology and inflammatory cytokine assessment. The results indicated that inhalation of BromAc^®^ over 28 days did not affect general health, behavior, or body weight compared to controls. No signs of respiratory distress, hemorrhage, hypoxia, or any other disorders were observed. Mild, non-dose-dependent lung pathology and inflammatory changes were observed across all groups including controls. There was no difference between treated and control groups on multiplex immunoassay. These findings suggest that BromAc^®^ is safe for inhalation at the tested concentrations and exposure duration.

##  Introduction

Respiratory diseases, including asthma, chronic obstructive pulmonary disease (COPD), and cystic fibrosis (CF), frequently result in increased mucus production due to inflammation and irritation of the airways. This inflammation triggers the body’s natural response to produce more mucus to trap and remove irritants such as dust, pollen, or smoke. In asthma, allergen exposure activates Th2 cells, which release cytokines, particularly IL-4, IL-5, and IL-13 [1]. In chronic bronchitis, long-term inflammation of the bronchi due to irritants like cigarette smoke or pollution activates immune cells, including neutrophils, lymphocytes, and macrophages which release inflammatory mediators such as IL-1, IL-6, and TNF-alpha [2]. In both asthma and chronic bronchitis, these mediators prompt goblet cells and submucosal glands to produce more mucus, while inflammation causes goblet cell hyperplasia and submucosal gland hypertrophy, resulting in thick, sticky mucus that blocks the airways [3]. In cystic fibrosis, the mucus is much thicker and stickier than normal, which can also block airways and lead to severe respiratory problems. Studies have shown that the viscosity of cystic fibrosis mucus is not necessarily greater than that of mucus in chronic bronchitis or asthma, but its dehydrated gel matrix and higher concentration of mucins contribute to its thickness and stickiness [4].

Mucus is a complex, gel-like secretion primarily composed of water, but it also contains a variety of other important components that give it its protective and functional properties. High-molecular-weight glycoproteins (mainly MUC5AC and MUC5B in the airways) provide the gel-like, viscoelastic properties of mucus, making up about 0.5–3% of its composition [4]. About 0.9–1% of mucus consists of salts (such as chlorides and bicarbonates of sodium and potassium), which help maintain its hydration and pH. Globular proteins (about 1.1%) include enzymes (lysozymes), immunoglobulins (especially IgA), and other antimicrobial or anti-inflammatory proteins that help to neutralize or destroy pathogens trapped within it. Lipids contribute to the barrier function and surface tension properties of mucus, making up about 0.3–0.5%. Especially during infection or inflammation, mucus may also contain DNA from dead cells, bacteria, with other cellular debris [5, 6].

Mucus acts as a critical defense mechanism in the respiratory tract. It traps inhaled irritants, such as dust, allergens, bacteria, and viruses, preventing them from penetrating deeper into the lungs and causing infection or damage. The mucus layer, together with the movement of cilia helps transport trapped particles out of the lungs, either by moving them toward the throat to be swallowed or expelled through coughing [7]. If cilia are damaged (by infection, smoking, or genetic diseases like primary ciliary dyskinesia), or if their movement is uncoordinated, mucus clearance is impaired, making it harder to remove mucus from the respiratory tract [8].

For mucus to be effectively moved by cilia, it must have physiologically optimal viscosity and hydration. When mucus becomes too thick or sticky, as seen in conditions like asthma, chronic bronchitis, or cystic fibrosis, it resists movement and adheres to the airway walls. In many respiratory diseases, inflammation overwhelms the mucociliary system’s ability to clear mucus by triggering excessive mucus production. This leads to further complications and impairs respiratory function [9].

Bromelain, a complex mixture of proteolytic enzymes, breaks down the protein structure of mucins, the main components of mucus, thereby dissolving thick, sticky secretions that are resistant to normal mucociliary clearance [10]. In a murine model of asthma, oral bromelain significantly reduced airway hyperreactivity, eosinophil infiltration, and IL-13 levels. It also decreased Bronchial Alveolar Lavage (BAL) CD19 + B cells and CD8 + T cells, demonstrating Bromelain’s anti-inflammatory effects [11, 12].

Nebulized Acetylcysteine (NAC) is a mucolytic agent that breaks down disulfide bonds in mucin proteins, significantly reducing the viscosity and elasticity of mucus [13]. Clinical studies and systematic reviews confirm that nebulized NAC thins and dissolves mucus, improving symptoms in conditions like COPD, asthma, bronchiectasis, and cystic fibrosis [13]. In patients with chronic respiratory diseases, nebulized NAC has been shown to significantly improve phlegm symptoms and enhance mucus clearance, as demonstrated by reduced phlegm scores in clinical trials [14, 15].

BromAc^®^ (MP-171) a new biologic containing modified, purified and fractionated protease derived from bromelain combined with NAC has been shown in multiple respiratory studies to be considerably more potent than its single component agents alone in dissolving respiratory mucus. The potent efficacy of BromAc^®^has been attributed to its dual action on both protein and disulfide linkages [16]. In ex-vivo studies using tracheal aspirates from critically ill patients, BromAc^®^ demonstrated a robust, dose-dependent mucolytic effect, with over 80% flow-through recovery at higher concentrations, compared to much lower rates for NAC alone [17]. The combination was reported to be up to 90 times more effective than NAC by itself in liquefying and clearing thick, immobile mucus [17]. In an artificial sputum and airway model representative of conditions like cystic fibrosis, COPD, and severe COVID-19, BromAc^®^ outperformed single-agent treatments [18]. The combination not only improved mucus flow but also visually and quantitatively dissolved mucus plugs that resisted standard mucolytic therapy [16–18]. In an ex-vivo ovine lung model, BromAc^®^ effectively dissolved mucus plugs and significantly reduced airway resistance compared to saline, with superior mucolytic activity over individual agents [19]. The particle size of nebulized BromAc^®^ is comparable to normal saline and suitable for airway delivery, and its mucolytic action is especially valuable in settings where mucus plugging leads to life-threatening airway obstruction, such as in ventilated patients or those with severe muco-obstructive diseases. Evaluating safety in animal models is a vital prerequisite for human clinical trials. The purpose of this study was to evaluate extended subacute short-term exposure safety of nebulized BromAc^®^.

## Materials and methods

### Drug Preparation

BromAc^®^ (MP-171) was manufactured by Mucpharm Pty Ltd., Australia. The investigational product was prepared in 0.9% saline and stored at -30 °C. The pH of the product was 6.97, osmolality 308 mOsm/L, and viscosity 1.075 mPa·s. The drug was thawed immediately before use and administered at ambient temperature. BromAc^®^ at 0.250/20 mg/mL has been tested previously in vitro and ex vivo [16, 18–20]. In this safety in vivo study, BromAc^®^ 0.250/20 mg/mL, along with 1-fold and 2-fold higher concentrations, was prepared to investigate any evidence of a safety limit of BromAc^®^ inhalation.

### Safety of nebulization BromAc^®^ in long-term safety mouse model

**Fig. 1:**
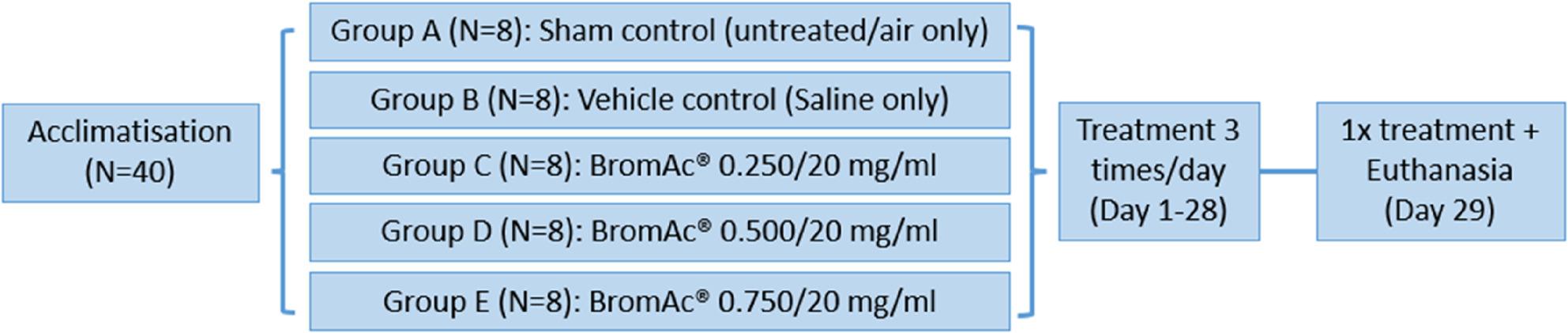
Overview of the design of the safety study. The study using 40 BALB/c mice (ACEC approval # iRECS6785) was conducted with five treatment groups: Sham control (Group A), Saline only (Group B) BromAc® 0.250/20 mg/mL (Group C), BromAc® 0.500/20 mg/mL (Group D), and BromAc® 0.750/20 mg/mL (Group E). All groups received 3 treatments per day for 28 days. On 29th day all groups received one treatment which was followed by euthanasia. Blood, tissue, and bronchoalveolar lavage samples were collected for analysis. The study aimed to assess the short-term subacute safety of BromAc® inhalation

 Forty (40) BALB/c mice (7 weeks old), sourced from Ozgene ARC (Perth, WA, AUS), were used in this inhalation study to assess the safety of BromAc^®^. Upon arrival at the animal facility, mice were acclimatized for at least one week prior to study commencement. The study consisted of 5 groups (*n* = 8 per group; 4 males and 4 females) (Fig. 1). Group A served as the sham control (untreated/air only), exposed to the nebulizer procedure without any drug. Group B was the vehicle control (saline only). Groups C, D, and E received BromAc^®^ at concentrations of 0.250/20, 0.500/20, and 0.750/20 mg/mL, respectively.

Each group received inhalation treatment 3 times daily for 28 days (Days 1–28), with a final single dose administered on Day 29. Each exposure involved a 10-minute inhalation session in a whole-body inhalation chamber connected to a Philips Respironics InnoSpire Elegance Nebulizer (Philips, Amsterdam, NLD).

Animals were monitored three times daily, 30 min after each treatment, with 2 hours between doses. Criteria for early euthanasia included a category score of ‘0’, ≥ 20% body weight loss, or evidence of hemorrhage (skin, eye, nose, anal, subcutaneous).

On Day 29, animals were euthanized by intraperitoneal injection of sodium pentobarbitone (60 mg/kg in 0.9% saline), followed by exsanguination via cardiac puncture for blood collection. Euthanasia has been performed IP injection instead of inhalation methods to preserve the lung tissue for subsequent analyses. Post-euthanasia, the lungs and internal viscera samples were collected for analysis.

### Histopathology

Formalin-fixed, paraffin-embedded sections were stained using H&E and Periodic Acid-Schiff (PAS) standard techniques. Images were captured using a binocular light microscope (Axioskop, Zeiss, GER) with a digital camera (Axiocam 506 Color, Zeiss, GER).

### Multiplex immunoassay

Cytokines were analyzed using the Milliplex MAP Mouse Th17 Panel, kit (Cat # MTH17MAG-47 K; Merck KGaA, Lot number 4200203, Darmstadt, GER). The kit contained the following analytes: IL-25/IL-17E, GM-CSF, IFN-y, IL-1b, IL-4, IL-5, IL-6, IL-17(p70), IL-13, IL-33, and TNF-a. The assay was conducted according to the manufacturer’s instructions. All samples were analyzed manually upon a 96-well plate, and the plate was washed on Bio-Plex Pro II magnetic plate washer (Bio-Rad, Hercules, CA, USA) x and read with the Bio-Plex Systems 200 (Bio-Rad, Hercules, CA, USA). Primary incubation was performed at 4 °C, with shaking, overnight. During subsequent incubations, the assay plate was shaken at 600 rpm at 25 °C and protected from light. Samples were analyzed and standard curves generated using the Bio-Plex Manager v6.0 software (Bio-Rad, Hercules, CA, USA).

### Statistical analysis

Statistical analysis was performed using GraphPad Prism version 10 (GraphPad Software, Inc., San Diego, California, USA). Repeated measures Two-way ANOVA has been used to analyze clinical score and body weight data. One-way ANOVA has been used to analyze cytokines multiplex data.

## Results

### General health and behavioral observations

**Fig. 2:**
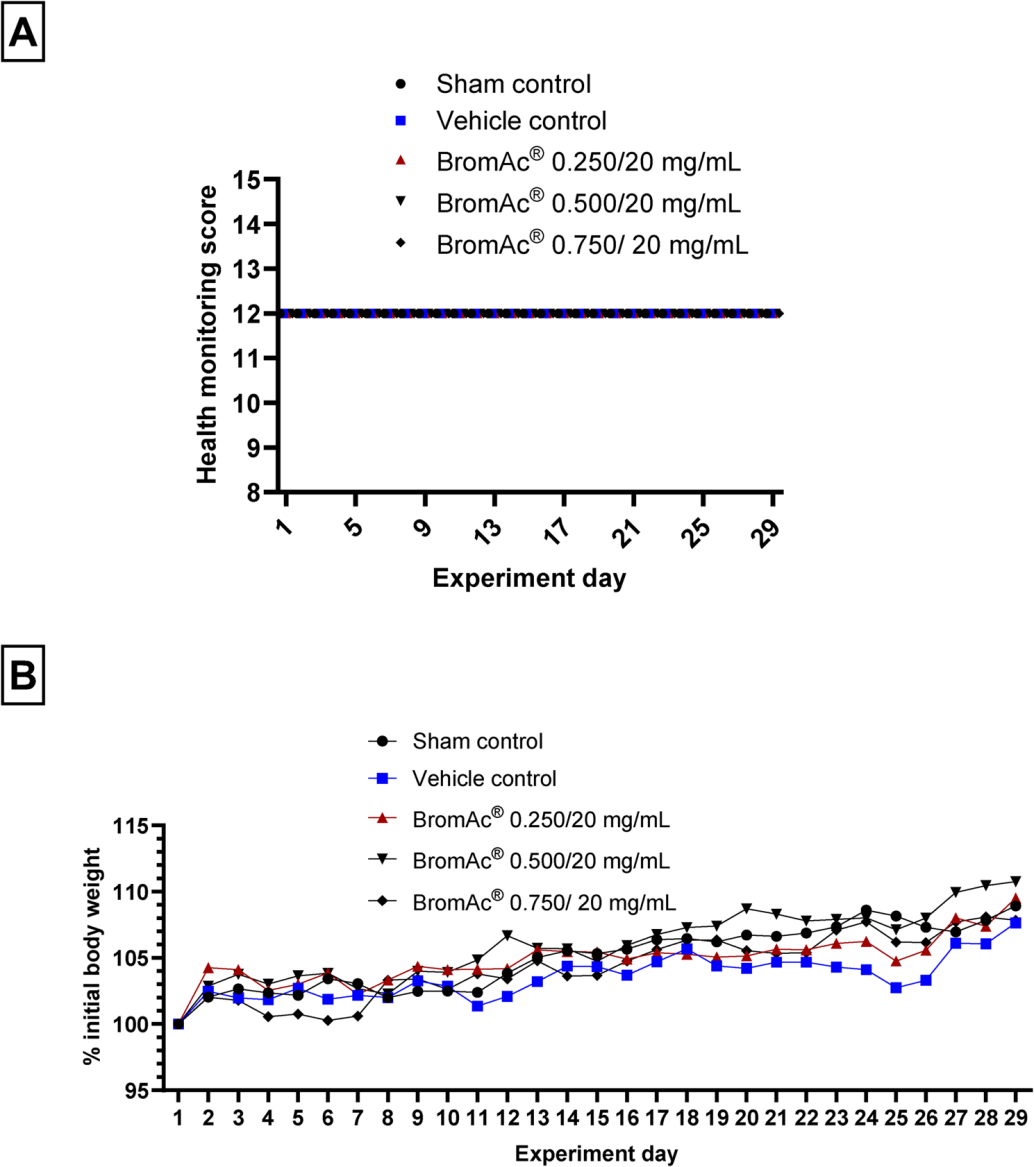
**A** Health monitoring score of animals in each group. **B** Mean body weight fluctuation of animals in each group showing the long-term safety of administration of BromAc® via inhalation

Following inhalation treatments, all mice maintained normal activity levels, including regular food and water intake, as well as normal respiratory patterns. General health monitoring scores of the mice, which were assessed based on parameters such as general wellbeing, pain, and distress, including “Appearance,” “Body condition,” “Natural behavior,” and “Provoked behavior”, revealed no adverse effects attributed to BromAc^®^ (Fig. 2A). Furthermore, there were no signs of hemorrhage, hypoxia, or respiratory distress in any of the control or treated mice. Additionally, there was no significant change in body weight fluctuations throughout the treatment course between treated and control group (Fig. 2B).

### Histopathological findings

**Fig. 3:**
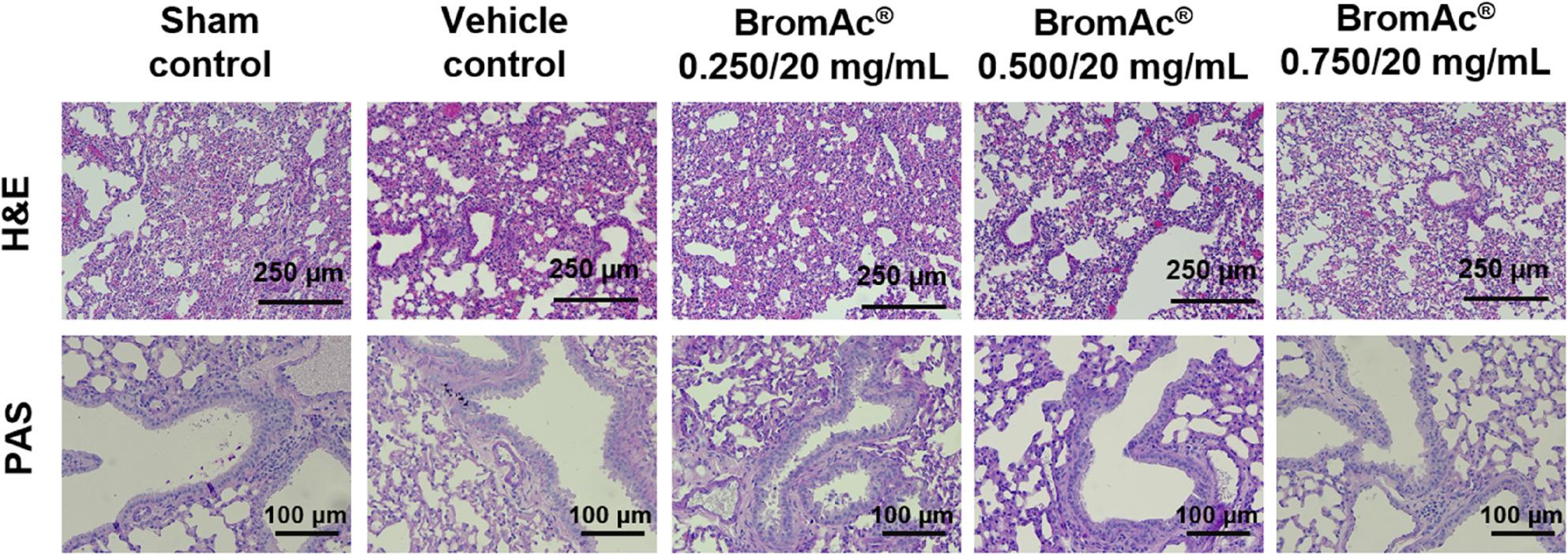
Representative histological images from BromAc® long term inhalation chamber safety study showing livers, kidneys, pancreases, intestine, spleen, and heart sections stained with H&E. Scale bar: 250 μm (magnification, ×100). Histological analysis revealed no signs of toxic alterations in the examined tissues of mice treated with BromAc® at either 0.250/20, 0.500/20, or 0.750/20 mg/mL, compared to the control groups

**Fig. 4:**
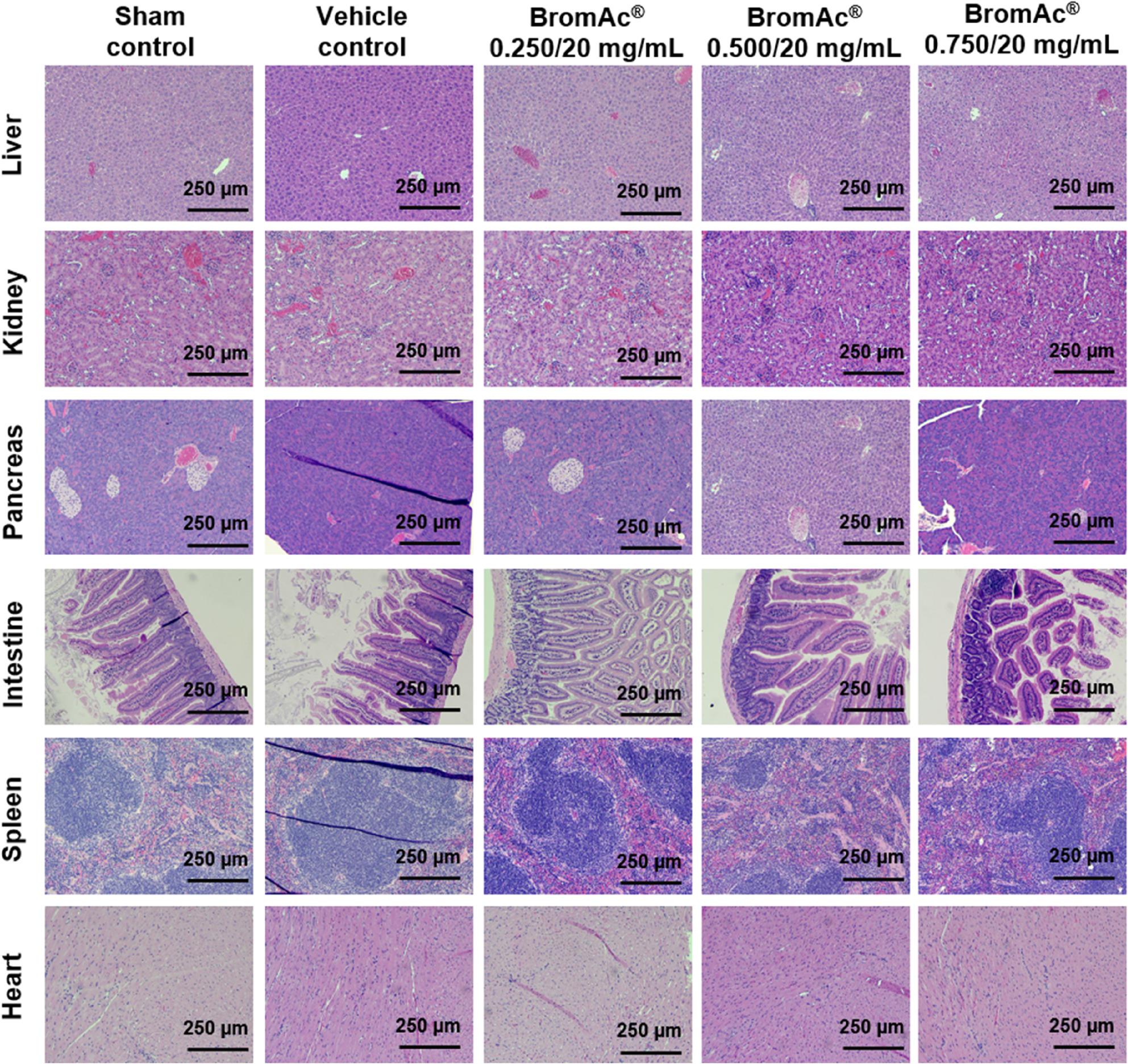
Representative histological images of lungs sections from BromAc® long term inhalation chamber safety study. Upper panel: lung sections stained with H&E. Scale bar: 250 μm (magnification, ×100), showing low severity of histopathological findings across all experimental cohorts, including controls, supports their classification as incidental, or artifactual rather than as direct effects of the experimental intervention. Lower panel: lung sections stained with PSA. Scale bar: 100 μm (magnification, ×200), showing absence of bronchiolar goblet cell hyperplasia and intra-airway mucus in both treated and control groups

Mild lung pathology was observed in some animals across all groups, including controls and treatment cohorts (Fig. 3; Table 1). Notable findings included increased alveolar macrophages and, rarely, multinucleated giant cells observed in the sham control (2/8), vehicle control (2/8), low dose (1/8), and medium dose (1/8) groups. Mild perivascular inflammatory infiltrates were commonly observed in many cases however were classified as background findings due to minimal severity. Artefactual hemorrhage was frequently noted in all groups. Importantly, no evidence of bronchiolar goblet cell hyperplasia or intra-airway mucus was observed in any group (Fig. 4). Mild inflammation of the right atrioventricular and/or right ventricular wall, possibly representing early epicardial mineralization, was seen in a small number in the sham control (3/8) and low dose (1/8) groups. Liver changes appeared to be unrelated to the treatment and were likely due to dietary or strain factors.

**Table 1:** Shows the pathological findings in mice of the long-term inhalation safety study. Results shows number of animals out of 8. Mice were either untreated (sham control) or treated with 0.9% NaCl (vehicle control), BromAc^®^ 0.250/20 mg/mL, BromAc^®^ 0.500/20 mg/mL, or BromAc^®^ 0.750/20 mg/mL

Histopathological Findings	Sham control	Vehicle control	BromAc^®^ 0.250/20 mg/mL	BromAc^®^ 0.500/20 mg/mL	BromAc^®^ 0.750/20 mg/mL
Lung					
Mild diffuse perivascular inflammatory cell infiltration	3/8	3/8	2/8	6/8	5/8
Mild multifocally within alveolar spaces are macrophages and rarely multinucleated giant cells	2/8	2/8	2/8	1/8	-
Mild multifocal alveolar and/or bronchiolar hemorrhage	7/8	6/8	3/8	7/8	3/8
Heart					
No abnormalities	5/8	7/8	7/8	8/8	8/8
Multifocal subepicardial mixed inflammatory infiltration in the right ventricle	3/8	-	1/8	-	-
Spleen					
No abnormalities	8/8	8/8	8/8	8/8	8/8
Liver					
No abnormalities	2/8	8/8	3/8	-	2/8
Diffuse hepatocellular microvesicular (glycogen-type) degeneration					
Mild	4/8	-	-	-	-
Moderate	1/8	-	-	-	-
Diffuse centrilobular hepatocellular glycogen-type microvesicular degeneration					
Mild	1/8	-	2/8	-	2/8
Moderate	-	-	1/8	4/8	1/8
Severe	-	-	-	1/8	1/8
Kidney, pancreas, intestines					
No abnormalities	8/8	8/8	8/8	8/8	8/8

### Inflammatory cytokine analysis

**Fig. 5:**
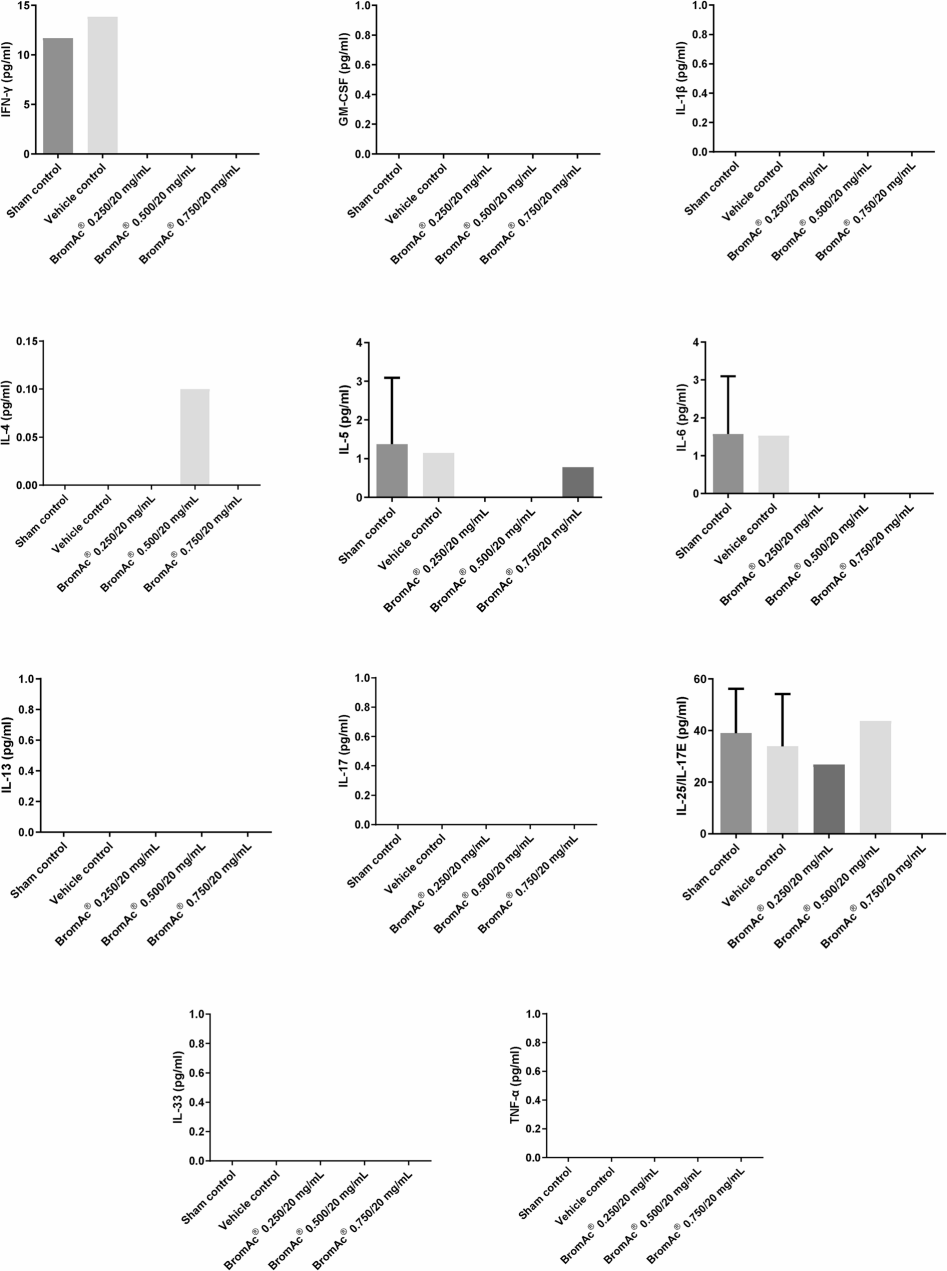
Multiplex cytokine analysis of Bronchial Alveolar Lavage (BAL) samples from the five treatment groups: Sham control, 0.9% NaCl Saline only, BromAc® 0.250/20 mg/mL, BromAc® 0.500/20 mg/mL, and BromAc® 0.750/20 mg/mL

Levels of key inflammatory cytokines (IL-1β, IL-4, IL-5, IL-6, IL-13, IL-17, IL-33, IL-25/IL-17E, GM-CSF, IFN-γ, and TNF-α) in BAL fluid were assessed using multiplex immunoassay (Fig. 5). One-way ANOVA statistical analysis revealed no significant differences in cytokine levels between the control and BromAc^®^-treated groups.

## Discussion

Extended, repeat dosing and long-term administration strategies for the treatment of mucus-producing respiratory diseases, particularly in animal models, remain central to the preclinical evaluation of therapeutic agents for chronic airway conditions such as COPD, chronic bronchitis, and cystic fibrosis. Chronic mucus hypersecretion has been identified as a primary contributor to morbidity and progression of these diseases due to its role in airway obstruction and promotion of recurrent infections [21]. Assessment of safety in animal models over extended periods is a critical step before advancing to human clinical trials, as it used to evaluate potential toxicities, and effects that may not be detected in single dose short-term investigations [22].

In the current study, subacute, repeat dose, 28 day short-term administration of BromAc^®^ (MP-171) did not show any adverse effects on the clinical score parameters, including appearance, body condition, natural behavior, and provoked behavior. Animals also maintained consistent and normal grooming, posture, and responsiveness across the extended administration period. These findings suggest that BromAc^®^ is well tolerated and safe for subacute use in the tested model. Such findings are consistent with prior reports that comprehensive behavioral and clinical evaluations in animal models serve as reliable indicators for drug safety during prolonged exposure periods [23]. In addition, BromAc^®^-treated groups did not show changes in body weight fluctuation compared to control groups. Stability in body weight is a widely recognized indicator of general health and tolerability in preclinical studies. Sustained loss or gain in body weight can reflect underlying adverse effects [24]. Hence, the absence of body weight alterations over this extended period supports the conclusion that BromAc^®^ is safe for progression to long-term administration and subacute settings. 

Mild perivascular infiltrates, observed with mild severity in control and treated cohorts, is widely acknowledged as an incidental finding in laboratory animals [25]. Their presence across all groups, irrespective of dosing, supports the view that these lesions are not compound-specific but represent common background pathology. Artefactual hemorrhage has similarly been recognized as a frequent artifact arising during tissue collection and processing, especially in the delicate pulmonary vasculature in mice. The absence of bronchiolar goblet cell hyperplasia and intra-airway mucus is significant, showing the lack of treatment-related secretory epithelial activation or significant chronic airway irritation. Such changes, if present, would indicate ongoing airway remodeling or allergic-type pathology, neither of which was identifiable in any group.

The increased number of alveolar macrophages, observed in both treated and control animals, likely reflect an adaptive response to repeated inhalation interventions, environmental exposures, or mild subclinical effect rather than a specific reaction to administration of BromAc^®^. The sporadic appearance of multinucleated giant cells is notable but not unique to the drug treatment cohorts. Their presence at low frequency, even in sham controls, is consistent with the responses previously documented in rodent and non-rodent species subject to chronic experimentation [26].

Localized mild inflammation of the right atrioventricular or ventricular myocardium, potentially indicative of early epicardial mineralization, was an uncommon finding observed at low numbers in the sham and low dose groups. Its lack of correlation with dose, alongside the unaffected medium dose and vehicle controls, indicate that these lesions are not attributed to the drug. Likewise, hepatic alterations were observed in both control and treated animals, interpreted as unrelated to the interventional groups exposed to BromAc^®^, with etiology likely attributable to background strain predispositions or diet, a common confounder in animal toxicology research.

After subacute, repeat dose, short-term administration of BromAc^®^ (MP-171) in the current animal safety study, One-way ANOVA statistical analysis revealed no significant differences in cytokine levels between the control and intervention groups. The results indicate repeated, prolonged exposure to BromAc^®^ did not induce changes in inflammatory cytokine expression, and that no adverse immune activation or suppression was attributed to BromAc^®^ inhalation exposure. Such findings provide valuable preclinical evidence supporting the safety profile of BromAc^®^ for extended use in safety animal models. In separate studies, BromAc^®^ effectively modulated immune cell populations in response to SARS-CoV-2, reducing CD16 + neutrophils and CD14 + monocytes while enhancing HLA-DR expression in monocytes. It also decreased TNF-a production by CD19 + B-cells, indicating improved immune response regulation [27]. In addition, in tracheal aspirates from critically ill COVID-19 patients ex-vivo, BromAc^®^ showed anti-inflammatory effects, reduced the activity of chemokines like MIP-1alpha, CXCL8, MIP-1b, MCP-1, and IP-10, as well as regulatory cytokines IL-5, IL-10, IL-13, IL-1Ra, and IL-9 [17]. In an Ovalbumin-induced mice model, BromAc^®^ nebulisation significantly reduced levels of IL-4, IL-5, and GM-CSF in BAL [28]. This differential effect between the BromAc^®^ effects in safety and disease models suggests that while BromAc^®^ actively modulates immune pathways relevant to the disease, it does not provoke unintended systemic cytokine effects under healthy conditions, supporting a favorable safety profile for BromAc^®^.

Each exposure method has its own limitations. In this study, we used a whole‑body inhalation chamber, which can lead to unintended deposition on chamber walls, fur, and oral surfaces. Therefore, accurately measuring the dose delivered to the lungs is technically challenging. To address this issue, measuring drug bioavailability in lung BAL fluid and plasma is required and is under assessment. Furthermore, translating animal safety data to humans for risk prediction is not always reliable, as false-positive or false-negative results may occur. Further long-term and progression to clinical safety trials in humans is necessary.

Collectively, these findings indicate that inhaled BromAc^® ^(MP-171) at the concentrations investigated over a 28-day duration, does not adversely affect the general health, behavior, or induce significant pulmonary or systemic toxicity in mice. The absence of significant changes in inflammatory cytokine profiles further confirms the safety of inhaled BromAc^®^ under the present experimental conditions.

## Data Availability

The datasets generated during and/or analyzed during the current study are available from the corresponding author on reasonable request.
